# Cellular Preoxygenation Partially Attenuates the Antitumoral Effect of Cisplatin despite Highly Protective Effects on Renal Epithelial Cells

**DOI:** 10.1155/2017/7203758

**Published:** 2017-02-19

**Authors:** Bahram Rasoulian, Ayat Kaeidi, Maryam Rezaei, Zahra Hajializadeh

**Affiliations:** ^1^Razi Herbal Medicines Research Center and Department of Physiology, Lorestan University of Medical Sciences, Khorramabad, Iran; ^2^Department of Physiology and Pharmacology, Rafsanjan University of Medical Sciences, Rafsanjan, Iran; ^3^Physiology-Pharmacology Research Center, Rafsanjan University of Medical Sciences, Rafsanjan, Iran; ^4^Razi Herbal Medicines Research Center, Lorestan University of Medical Sciences, Khorramabad, Iran

## Abstract

Our previous in vitro studies demonstrated that oxygen pretreatment significantly protects human embryonic renal tubular cell against acute cisplatin- (CP-) induced cytotoxicity. The present study was designed to investigate whether this protective effect is associated with decreasing therapeutic effects of cisplatin on malignant cells. For this purpose, cultured human embryonic kidney epithelial-like (AD293), cervical carcinoma epithelial-like (Hela), and ovarian adenocarcinoma epithelial-like (OVCAR-3) cells were subjected to either 2-hour pretreatment with oxygen (≥90%) or normal air and then to a previously determined 50% lethal dose of cisplatin for 24 hours. Cellular viability was evaluated via MTT and Neutral Red assays. Also, activated caspase-3 and Bax/Bcl-2 ratio, as the biochemical markers of cell apoptosis, were determined using immunoblotting. The hyperoxic preexposure protocol significantly protects renal AD293 cells against cisplatin-induced toxicity. Oxygen pretreatment also partially attenuated the cisplatin-induced cytotoxic effects on Hela and OVCAR-3 cells. However, it did not completely protect these cells against the therapeutic cytotoxic effects of cisplatin. In summary, the protective methods for reducing cisplatin nephrotoxic side effects like oxygen pretreatment might be associated with concurrent reduction of the therapeutic cytotoxic effects of cisplatin on malignant cells like cervical carcinoma (Hela) and ovarian adenocarcinoma (OVCAR-3) cells.

## 1. Introduction

Cisplatin (CP) (*cis*-diamminedichloroplatinum (II)) as an antineoplastic drug is a standard component in treatment regimens for many solid tumors like head and neck, ovarian, cervical, and testicular cancers. The efficacy of cisplatin is dose-dependent and the main dose-limiting side effect of cisplatin is nephrotoxicity. However, there are some other adverse effects like ototoxicity, neurotoxicity, gastrotoxicity, and myelosuppression [1, 2]. It is well known that cisplatin, depending on the dose of administration, causes renal tubular cell death at least through affecting two distinct cellular mechanisms. With higher doses, necrotic cell death is predominant, whereas lower concentrations which are most commonly used in real clinical practice primarily induce apoptosis [1, 3]. Even with hydration, as the accepted method for reducing cisplatin nephrotoxicity, about one-third of patients treated with this drug have some evidence of renal damage (such as transient elevation of blood urea nitrogen levels) within the days following cisplatin administration [4].

It has been demonstrated that oxygen pretreatment largely reduces cisplatin-induced nephrotoxicity both in vitro and in vivo [5–7] and some clinical studies have used hyperoxic pretreatment method for reducing cisplatin-induced nephropathy in cancer patients [8]. Nevertheless, it has not been revealed whether this highly protective effect of oxygen pretreatment on renal tubular cells is associated with reduced therapeutic cytotoxic effects of cisplatin on malignant cells. In fact, the present study focused on preclinical and in vitro aspects of this concern.

On the other hand, it has been proposed that normobaric or hyperbaric oxygen therapy elicits direct antitumoral effects [9] and in some cases can enhance chemotherapeutic activity of some anticancer drugs especially on hypoxic tumoral cells. For example, it has been shown that combined administration of cisplatin and hyperbaric oxygen enhances chemotherapeutic response to cisplatin in epithelial ovarian cancer cells in mice xenografts [10]. Despite the possible chemosensitizing effect of simultaneous oxygen administration, the direct effects of malignant cell “preoxygenation” on chemotherapeutic activity of anticancer drugs such as cisplatin have not been studied yet. It should be noted that, in the course of hyperbaric or hyperoxic exposure, unlike the inner cells of the tumor, superficial tumoral cells are exposed to higher tissue oxygenation. This in vitro study was designed to compare the effects of short course and single dose hyperoxic pretreatment on cytotoxic and apoptotic effects of cisplatin in normal renal tubular epithelial cells and two malignant ovarian (OVADR-3) and cervical (Hela) cell lines.

## 2. Materials and Methods

### 2.1. Materials

Cell culture reagents, fetal bovine serum (FBS), penicillin-streptomycin solution, and trypsin EDTA were purchased from Biosera Co. (East Sussex, UK). Dishes and culture flasks were obtained from SPL Lifesciences Inc. (Gyeonggi-do, South Korea). Cisplatin, 3-[4,5-dimethyl-2-thiazolyl]-2,5-diphenyl-2-tetrazolium bromide (MTT), and Neutral Red were acquired from Sigma (St. Louis, MI, USA). Primary polyclonal anti-caspase-3 and primary monoclonal anti-*β*-actin antibodies were purchased from Cell Signaling Technology, Inc. (Beverly, MA, USA). Primary polyclonal anti-Bax and primary monoclonal anti-Bcl-2 antibodies were purchased from Santa Cruz Biotechnology, Inc. (Delaware Ave., Santa Cruz, USA).

### 2.2. Cell Culture

AD293 (human embryonic kidney epithelial-like), Hela (human cervical carcinoma epithelial-like), and OVCAR-3 (human ovarian adenocarcinoma epithelial-like) cells were purchased from National Cell Bank of Iran (NCBI), Pasteur Institute of Iran (Tehran, Iran). Cells were grown on Dulbecco's modified Eagle's medium (DMEM) enhanced with 10% fetal bovine serum, penicillin (100 U/mL), and streptomycin (100 mg/mL). They were preserved at 37°C under an atmosphere of 5% CO_2_.

### 2.3. Experimental Groups

Four experimental groups were assessed for each cell type as follows: (1) cells incubated with normal air flow and cisplatin vehicle (Air + Veh); (2) cells incubated with oxygen flow and cisplatin vehicle (O_2_ + Veh); (3) cells incubated with normal air flow and cisplatin (Air + CP); (4) cells incubated with oxygen flow and cisplatin (O_2_ + CP).

**Figure 1:**
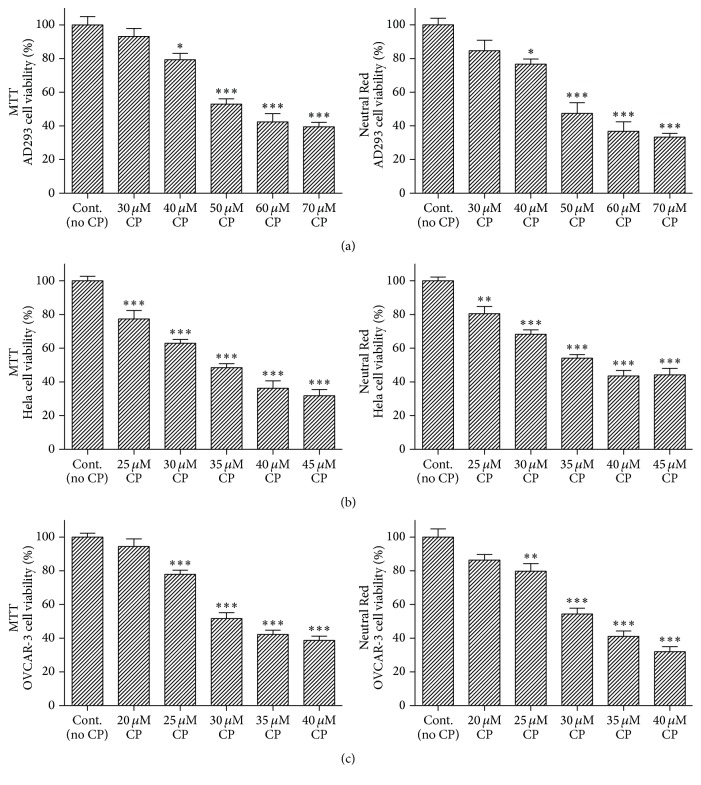
Effect of cisplatin on cell viability. Different cells types (AD293-(a), Hela-(b), and OVCAR-3-(c)) were incubated with variant doses of cisplatin for 24 h. Cell viability was determined by MTT and Neutral Red assays. Data are mean ± SD; *n* = 6–8 wells for each group/cell type; ^*∗*^*p* < 0.05, ^*∗∗*^*p* < 0.01, and ^*∗∗∗*^*p* < 0.001 versus control.

All three cell types (AD293, Hela, and OVCAR-3) were grown in plastic culture flasks and used in exponential growth phase or when grown to confluent monolayer. Growth medium was changed three times a week. For the MTT and Neutral Red assays, cells were plated at the density of 5000 per well in a 96-well microplate (7-8 wells were determined for each experimental group). In experiments related to each cell type, cells in “Air + Veh” and “O_2_ + Veh” groups were grown in normal medium, and cells in the other 2 groups were grown in culture medium with cisplatin. For protein extraction, cells were grown in a six-well plate and allowed to attach and grow for 24 h. Cisplatin was dissolved in phosphate-buffered saline (PBS), and then the cells were incubated in a medium with cisplatin or vehicle (PBS) for 24 h. Cisplatin was used by its previously determined lethal dose 50% (LD_50_) in experiments related to each cell type, that is, 50 *μ*M, 35 *μ*M, and 30 *μ*M for AD293, Hela, and OVCAR-3 cells, respectively. LD_50_ was approved by both MTT and Neutral Red cell viability assays (Figure 1).

### 2.4. Hyperoxic Pretreatment and Cisplatin Administration

Prior to drug treatment, to produce hyperoxia preexposure, different cell line plates in incubator were exposed to 95% oxygen/5% CO_2_ with continuously flowing humidified atmosphere for 2 h at 37°C. Parallel plates were kept in continuously flowing humidified 95% air/5% CO_2_. Closely following oxygen pretreatment, cisplatin (or vehicle) was added to the culture plates. Via an oxygen meter (Lutron DO-5510, Taiwan), oxygen content of the chamber was checked throughout the preexposure period. It should be noted that, initially, there were 2 other control groups in each experimental set in which cells were simply incubated with normal air and 5% CO_2_ without any flow. However, continuous air flow did not affect the cell viability or apoptotic markers. Thus, the control groups, treated with cisplatin or vehicle without any air flow, were not considered in the final analysis.

### 2.5. Cell Viability Analysis

*Neutral Red Assay.* For in vitro cellular cytotoxicity evaluation, Neutral Red assay has been widely used. This test is based on the combination of Neutral Red (3-amino-7-dimethyl-1-2-methylphenazine hydrochloride) into the lysosomes of viable cells. Neutral Red (4 mg/mL) was diluted into medium (1 : 100) and incubated overnight at 37°C and, before use, the Neutral Red solution was centrifuged. Prepared Neutral Red solution (200 *µ*L) was added to any well and the cells were incubated for 3 h (37°C). After that, using 1% calcium chloride and 0.5% formaldehyde solution, the cells were quickly washed. After 10 min incubation of the cells (at room temperature) with a 50% ethanol and 1% acetic acid solution, the Neutral Red dye was released from the viable cell. Finally, absorbance (OD) values were measured by spectrophotometry at 540 nm. Results values were expressed as percentages of control.

*MTT Assay.* Cellular viability was evaluated by the 2-(4,5-dimethylthiazol-2-yl)-2,5-diphenyltetrazolium bromide (MTT) reduction to formazan. MTT was dissolved in PBS and added to the culture media at 0.5 mg/mL as a final concentration. After additional 2 h incubation at 37°C, the supernatant media were carefully removed and 100 *µ*L DMSO was added to each plate well. Finally, wavelengths absorbance (OD) values were determined at 540 nm by spectrophotometry with a microplate reader apparatus (Eliza MAT 2000, DRG Instruments, GmbH). Each experiment was performed 5-6 independent times. Results were represented as percentages of control.

### 2.6. Western Blot Analysis

AD293, Hela, and OVCAR-3 cells were separately homogenized in ice-cold buffer containing 10 mM Tris-HCl (pH 7.4), 1 mM EDTA, 0.1% SDS, 0.1% Na-deoxycholate, and 1% NP-40 which is supplemented with protease inhibitors (1 mM sodium orthovanadate, 10 *µ*g/mL aprotinin, 1 mM phenylmethylsulfonyl fluoride, and 2.5 *µ*g/mL of leupeptin). The homogenate was centrifuged at 14000 rpm for 15 min at 4°C. The subsequent supernatant was preserved as the whole cell fraction. Via the Bradford method, protein concentration was evaluated. The same amounts of protein (40 *µ*g) were resolved on a 12% SDS-PAGE gel and finally transferred to PVDF membranes (Roche, Germany) electrophoretically. After overnight blocking (4°C) with 5% nonfat dried milk (blocking buffer, TBS-T, 150 mM NaCl, 20 mM Tris-HCl, and 0.1% Tween 20, pH 7.5), the PVDF membranes were explored with rabbit monoclonal antibody to caspase-3 (Cell Signaling Technology, 1 : 1000 overnight at 4°C) and Bax and Bcl-2 (Santa Cruz Biotechnology, 1 : 1000 for 3 h at room temperature).

The blots were incubated for 60 min (room temperature) by a secondary antibody conjugated to horseradish peroxidase (1 : 15000, GE Healthcare Bio-Sciences, USA) following washing three times in TBS-T. The blocking buffer was used as a diluent of antibodies. The complexes of antibody-antigen were indicated via the ECL system and exposed to chemiluminescent detection film (Roche, Germany). To analyze the intensity of the expression, Lab Work analyzing software (UVP, UK) was used. *β*-Actin immunoblotting (antibody from Cell Signaling Technology, USA; 1 : 1000) was used to control for loading. The western blot experiments for each protein were performed 4-5 independent times.

### 2.7. Statistical Analysis

Cell viability results were expressed as mean ± SEM. The difference in mean cell viability assays between experimental groups was examined by One-Way ANOVA, followed by Tukey's post hoc test. The values of protein band densities (Bax, Bcl-2, and caspase-3) were expressed as tested protein/*β*-actin ratio for each sample and were expressed as median (range) in related graphs. Different groups were compared by Kruskal-Wallis followed by Mann–Whitney* U* test between selected groups. GraphPad Prism (GraphPad Software, USA) and IBM SPSS Statistics (version 15) software were used for drawing graphs and statistical analysis, respectively. *p* < 0.05 was considered as significant.

## 3. Results

### 3.1. Cell Viability Results

**Figure 2:**
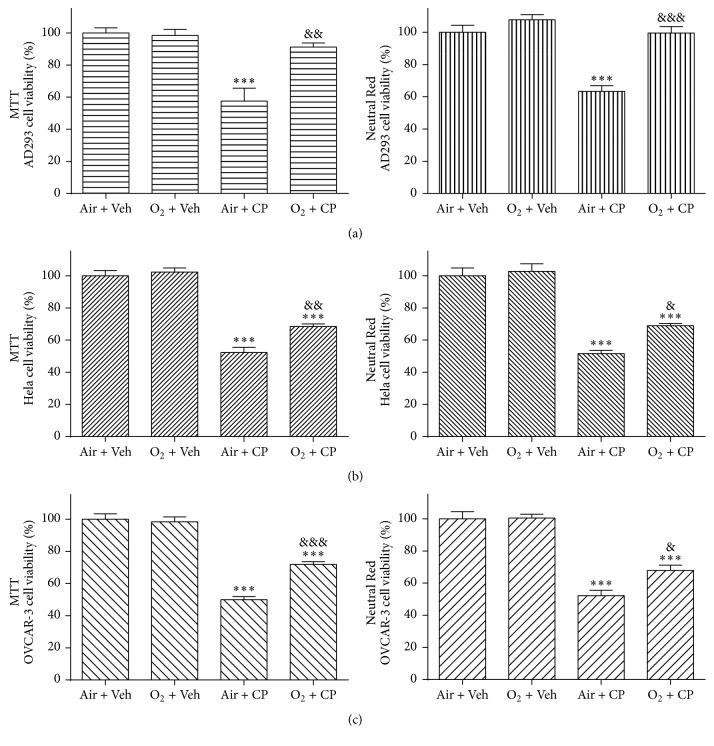
Hyperoxic preconditioning prevents cisplatin-induced cells death. AD293 (a), Hela (b), and OVCAR-3 (c) cells were treated with nearly pure oxygen (≥90%) preconditioning (2 h) and then cisplatin (50, 35, and 30 *μ*M for AD293, Hela, and OVCAR-3 cells, resp.) was added for an additional 24 h. Cell viability was then determined using the MTT and Neutral Red assays. Data are mean ± SEM; *n* = 6–8 wells for each group; ^*∗∗∗*^*p* < 0.001 versus both “Air + Veh” and “O_2_ + Veh” groups (in (a), (b), and (c) parts); ^&^*p* < 0.05, ^&&^*p* < 0.01, and ^&&&^*p* < 0.001 versus “Air + CP” group (in (a), (b), and (c) parts).

At first, we analyzed the effects of different concentrations of cisplatin on human embryonic kidney epithelial-like (AD293), cervical carcinoma epithelial-like (Hela), and ovarian adenocarcinoma epithelial-like (OVCAR-3) cells viability using the MTT and Neutral Red assays. After the initial 24 h attachment/growth period, confluent monolayers of cultured cells were exposed to cisplatin (at the concentrations of 20 to 70 *μ*M for 24 h). Figure 1 shows that cisplatin could decrease the viability of all cell types and this toxicity was dose-dependent. The toxic effect observed in 50, 35, and 30 *μ*M cisplatin for AD293, Hela, and OVCAR-3, respectively, resulted in approximately 50% decrease of relative cell viability and this was used as the optimum dose for damaging the cells and evaluating the protective effects of the oxygen pretreatment (Figure 2).

As shown in Figure 2, there was no significant difference between “Air + Veh” and “O_2_ + Veh” groups in any cell type indicating that 2 hours of 90% oxygen pretreatment did not have any toxic effect either on AD293 or malignant cells (Hela and OVCAR-3) (Figure 2). In AD293 cells, there was no significant difference among “O_2_ + CP” group and both vehicle treated groups and there was a significant difference between “O_2_ + CP” and “Air + CP” groups. Thus, MTT and Neutral Red assays showed that oxygen pretreatment largely protects human renal AD293 cells against acute single dose cisplatin-induced toxicity (Figure 2).

In contrast to normal AD293 cells, in the case of malignant Hela and OVCAR-3 cell lines, there was a significant difference between “O_2_ + CP” and both “Air + CP” and “O_2_ + CP” groups. This means that cisplatin cytotoxic effects on these cell lines exist despite hyperoxic pretreatment. But there was a significant reduction in the cytotoxic effects of cisplatin as determined by significant higher cell viability results in “O_2_ + CP” group compared to “Air + CP” group in both MTT and Neutral Red assays of these two cell lines (Figure 2).

### 3.2. Western Blot Results

**Figure 3:**
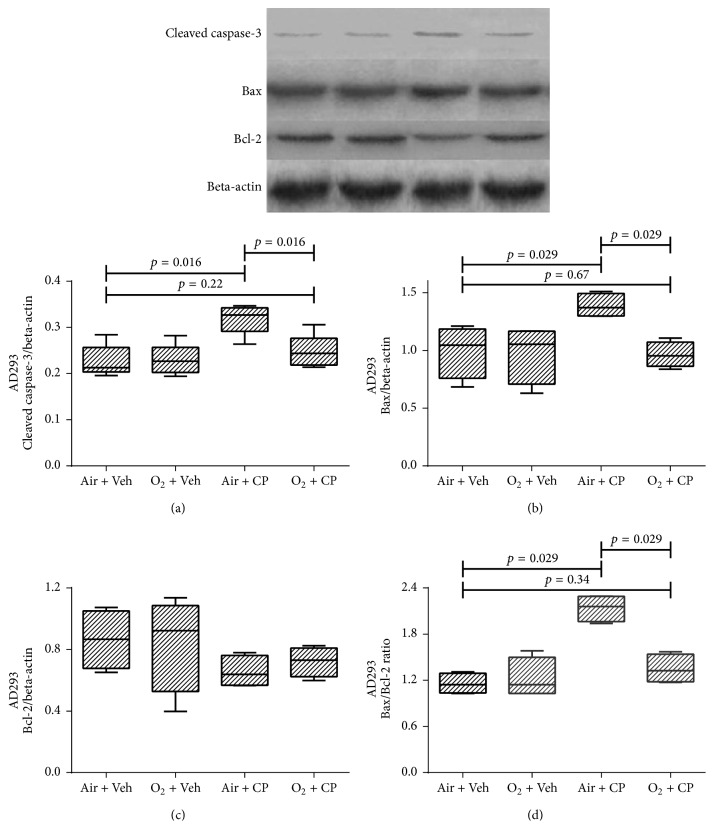
Western blot analysis of the caspase-3 protein activation, Bax, Bcl-2, and Bax : Bcl-2 ratio of AD293 cells. Cells were exposed to cisplatin (50, 35, and 30 *μ*M for AD293, Hela, and OVCAR-3 cells, resp.) and cisplatin plus hyperoxic preconditioning (2 h) for 24 h. Each value in the graph represents the mean ± SEM band density ratio for each group. Beta-actin was used as an internal control (*n* = 4).

**Figure 4:**
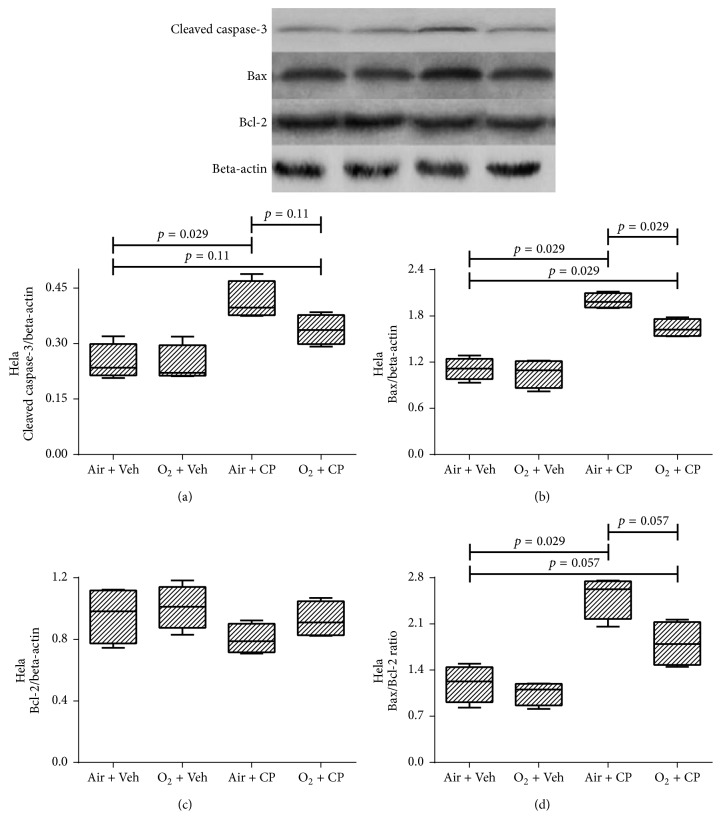
Western blot analysis of the caspase-3 protein activation, Bax, Bcl-2, and Bax : Bcl-2 ratio of Hela cells. Cells were exposed to cisplatin (50, 35, and 30 *μ*M for AD293, Hela, and OVCAR-3 cells, resp.) and cisplatin plus hyperoxic preconditioning (2 h) for 24 h. Each value in the graph represents the mean ± SEM band density ratio for each group. Beta-actin was used as an internal control (*n* = 4).

**Figure 5:**
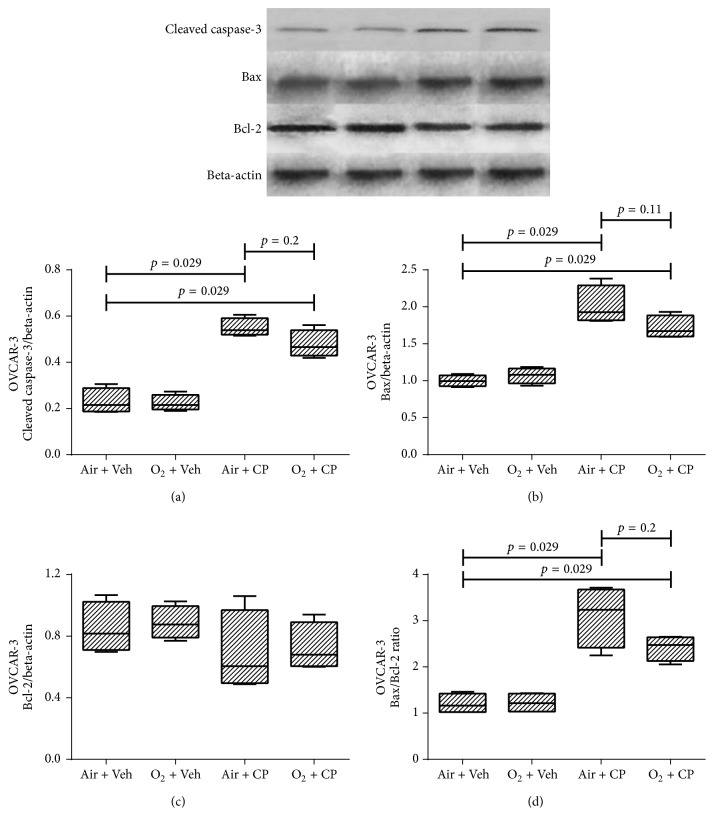
Western blot analysis of the caspase-3 protein activation, Bax, Bcl-2, and Bax : Bcl-2 ratio of OVCAR-3 cells. Cells were exposed to cisplatin (50, 35, and 30 *μ*M for AD293, Hela, and OVCAR-3 cells, resp.) and cisplatin plus hyperoxic preconditioning (2 h) for 24 h. Each value in the graph represents the mean ± SEM band density ratio for each group. Beta-actin was used as an internal control (*n* = 4).

Figure 3 shows western blot results of renal AD293 cells. It is obvious that cisplatin led to increased expression of apoptosis markers, that is, cleaved caspase-3, Bax, and Bax/Bcl-2 ratio. Furthermore, oxygen pretreatment significantly decreased cisplatin-induced apoptosis. There was not any significant difference in the expression level of the antiapoptotic protein, Bcl-2, among various groups in all 3 cell types (Figures 3–5). Also, cisplatin led to a significant higher expression of apoptotic markers in Hela and OVCAR-3 cells and there was a significant (or marginally significant) cisplatin-induced increase in expression of apoptotic markers, despite pretreatment with oxygen, except for the cleaved caspase-3 of Hela cells (*p* = 0.11 between “O_2_ + CP” and “Air + CP” groups, Figure 4(a)). There was a significant (Bax in Hela cells, Figure 4(b)), marginally significant (Bax/Bcl-2 in Hela cells, Figure 4(d)), or nonsignificant (cleaved caspase-3 in Hela cells (Figure 4(a)) and all apoptotic markers in OVCAR-3 cells (Figures 5(a)–5(d))) reduction of apoptotic markers in oxygen pretreated groups subjected to cisplatin administration.

### 3.3. Discussion

As mentioned in the Results, oxygen pretreatment attenuates, at least, some antitumor properties of cisplatin on Hela and OVCAR-3 cell lines; however, it is noteworthy that in this study the antitumor properties of cisplatin were not fully abolished. Cisplatin cellular toxicity on renal tubular AD93 cells was highly reduced in two control groups (with no cisplatin treatment) after oxygen preconditioning procedure. Bax and cleaved caspase-3 expiration, proteins related to apoptosis, were elevated fallowing cisplatin treatment in AD93 cells. Hyperbaric oxygen pretreatment elicited a significant inhibitory effect on elevated Bax and cleaved caspase-3 expiration on AD93 cells. It should be noted that cisplatin oxygen pretreatment before cisplatin therapy has no significant effect on Bcl-2 expiration in AD93 cells. Cisplatin increased caspase-3 activation and Bax expression as apoptosis markers in Hela cells. It should be noted that cisplatin-induced cellular toxicity, through apoptosis mechanisms, remained after oxygen preconditioning. In addition, after oxygen preconditioning, reduction in Bax expression was significant compared to cisplatin treated Hela cells (without oxygen preconditioning). But there was no significant deference in reduction of cleaved caspase-3 expressions between oxygen preconditioning + cisplatin and non-oxygen preconditioning + cisplatin treated Hela cells. Cisplatin treatment had no significant effect on Bcl-2 expression level in Hela cells. Cisplatin elevated Bax expression and caspase-3 activation in ovarian cancer cell line, OVCAR-3. Similar to Hela cells, cisplatin-induced apoptosis factors still remained following oxygen preconditioning. It should be noted that, following oxygen preconditioning + cisplatin, no significant difference was observed between the expression levels of Bax and cleaved caspase-3 compared to that of cisplatin treated OVCAR-3. Also, cisplatin therapy or oxygen preconditioning had no significant effect on Bcl-2 protein expression in OVCAR-3 cells.

As we know, cisplatin is one of the most potent antitumor platinum based agents. It is also a very effective compound against a wide spectrum of cancers [1]. Despite the useful properties of platinum compounds, they are toxic. Patients getting these agents experience strict side effects which in turn seriously restricts further administration. Thus, in order to achieve success in tumor chemotherapy, management of such drug-induced cytotoxicity is of critical significance. The side effects of platinum therapy include general cell-damaging effects, such as nausea, vomiting, and decreased number of blood cells and platelets, as well as reduced bone marrow production and attenuated response to infection. More specific side effects include damage to the kidney, neuronal damage, and hearing loss [11–13].

In this regard, the main controlling approaches include renoprotection and enhancing drug removal via hydration using osmotic diuretics. However, avoiding the nephrotoxic medications is crucial and additional therapies are also required.

Cisplatin causes significant oxidant loading to the renal epithelial cells through free radical production which results in damage to the antioxidant defense systems [14]. Cisplatin-induced oxidative stress has been proposed as an inducer in both Fas-mediated [15] and mitochondrial pathways [16, 17] of renal cell apoptosis [1].

Previous studies by this group have demonstrated that pretreatment with hyperbaric oxygen could protect the rat kidney against cisplatin-induced nephropathy [7]. In addition, it has been shown that oxygen preconditioning can protect human renal tubular cells from cisplatin-induced cytotoxicity in vitro [5]. Likewise, in animal models, oxygen pretreatment reduces ischemia-reperfusion injuries in various vital organs such as the central nervous system [18–21], liver [22, 23], heart [24], and kidney [25, 26]. Moreover, it has been reported that hyperoxic preconditioning could attenuate hypoxia-induced apoptosis in cultured mesenchymal stem cells [27]. In addition, the negative properties of deceased-donor hypoxia and useful properties of living-donor hyperoxic preconditioning on kidney graft function have been demonstrated in some clinical investigations [28, 29]; however, some others failed to show such a relevance [30].

Short-term pretreatment with oxygen partially elevated ROS induction in various tissues [31]. It seems that these useful effects are associated with the upregulation of endogenous mechanisms underlying cellular defense such as antioxidant systems and heat shock proteins [6, 21, 32]. On the other hand, a prior investigation revealed that intermittent oxygen exposure can induce more potent degrees of tolerance to ischemia in rat brain in comparison with continued oxygen pretreatment [19]. Furthermore, delayed cardioprotective effects of hyperoxic preconditioning against ischemia-reperfusion injury could be continued via intermittent oxygen administration [33]. Although long-term hyperoxic treatment could be toxic by itself, short-term oxygen preconditioning is a safe procedure that could be effortlessly applied in clinical practice [34].

As previously mentioned, cisplatin administration leads to significant oxidant loading to the renal epithelial cells through formation of free radicals as well as damage to cellular antioxidant defense systems. This explains, at least partially, the cellular mechanisms through which cisplatin-induced cytotoxic effects are mediated on renal tubular cells [35, 36].

Previous in vivo investigations have shown that short-term pretreatment with nearly pure oxygen causes some degrees of protection against nephrotoxicity induced by cisplatin and renal as well as cardiac ischemic injuries [5–7, 33]. These protective properties could be due to exciting the endogenous defense mechanisms such as antioxidant systems via induction of mild oxidative stress by hyperoxia [33]. However, there is no evidence in the literature in support of cisplatin-induced injury attenuation via oxygen pretreatment in human renal tubular cells.

An additional important concern is that most cisplatin cytotoxic effects are mediated via various common pathways among tumoral and renal epithelial cells. Hence, approaches that decrease cisplatin-induced nephropathy might have some unsought complications which in turn reduce the antitumor effect of this valuable drug [2].

Over the past half century, hyperbaric oxygen treatment has been used as an operative and safe cure for a variety of nonmalignant situations like decompression sickness, arterial embolism, and severe carbon monoxide poisoning [37, 38]. In addition, hyperbaric oxygen has been applied for the management of several chronic radiation injury forms [39–41]. A significant interpretation of the literature on the efficacy of hyperbaric oxygen treatment in this context is complicated by the heterogeneity of the treated disease, the various tissue damage types, and the several toxicity scoring systems used. Few randomized controlled trials using oxygen therapy for the management of chronic radiation injury have been investigated; however, the findings appear promising for some subgroups such as head and neck patients and for those with proctitis as a result of radiation [39].

Apoptosis investigations in neoplasms treated with hyperbaric oxygen are very limited. Two in vitro investigations on oral and breast cancer cells showed no significant change in apoptosis following hyperbaric oxygen [42, 43]. In addition, another study supports the activation of the proapoptotic pathway, mitogen-activated protein kinase (MAPK), and downregulation of the antiapoptotic pathway, extracellular-signal-regulated kinases (ERK), in hematopoetic cells following hyperbaric oxygen treatment [44].

Moreover, hyperbaric oxygen treatment has been shown to induce apoptosis in osteosarcoma cells [45]. Also, two different in vivo models, gliomas and breast tumors, have reported the induction of cell death following hyperbaric oxygen treatment [46–48].

Altogether, this may suggest that changes in concentration of oxygen affected the cellular antioxidant pathways [49], leading to a change in cell survival signaling. However, the map is multifaceted and mechanistic investigations are essential before any last conclusions can be drawn. Preexposure to oxygen with optimum method has considerable results in reducing cisplatin-induced renal injury in experimental studies and the results are largely encouraging for designing additional clinical trials in cancer patients.

Additional studies are required to investigate the protective effects of oxygen pretreatment against cisplatin-induced cytotoxicity among different tumoral and renal cells.

## 4. Conclusion

Hyperbaric oxygen preconditioning induces potent protective effects against cisplatin-induced renal epithelial cells toxicity. This protective effect may relate, at least in part, to a reduction in cisplatin-induced cellular apoptosis mechanisms. In spite of the potent protective effects, hyperbaric oxygen preconditioning may reduce the antitumoral properties of cisplatin. The cellular mechanisms underlying these effects may relate to a reduction in apoptosis factors.
